# Fabrication and characterisation of Ti and DLC coatings on metamaterial-architecture-inspired 3D-printed polymer substrates

**DOI:** 10.1007/s00170-026-17551-6

**Published:** 2026-02-02

**Authors:** Ely Dannier V-Niño, José Luis Endrino, Andrés Díaz Lantada, Iván Fernández Martínez, Hugo Armando Estupiñán Duran, Saurav Goel

**Affiliations:** 1 Materials Science, Engineering and Nanotechnology Researchers Network, Foundation of Researchers in Science and Technology of Materials, Bucaramanga, 680003 Colombia; 2 Nano4Energy SL, Alcalá de Henares, 28806 Spain; 3 Materials and Sustainability group, Universidad Loyola Andalucia, Sevilla, 41704 Spain; 4 Departamento de Ingeniería Mecánica, Universidad Politécnica de Madrid, Madrid, 28006 Spain; 5 Departamento de Materiales y Minerales, Universidad Nacional de Colombia, Medellín, 050034 Colombia; 6 School of Engineering and Design, London South Bank University, London, SE1 0 AA UK; 7 School of Advanced Engineering, University of Petroleum and Energy Studies, Dehradun, 248007 India

**Keywords:** Architected surfaces, Microtextured polymer substrates, Surface functionalization, Stereolithography laser technique, Photoreactive resins, Magnetron sputtering

## Abstract

This research explores the fabrication and characterisation of metamaterial-architecture-inspired 3D-printed polymer substrates with complex geometries, subsequently functionalized with titanium (Ti) and diamond-like carbon (DLC) coatings deposited by direct current magnetron sputtering. In this context, the term *metamaterial-architecture-inspired* refers exclusively to the engineered surface geometry and does not imply the experimental demonstration of emergent metamaterial properties. Polymeric substrates were fabricated via laser stereolithography using both an industrial (SLA-3500) and a low-cost (Form 1+) printing system, employing photoreactive resins in a layer-by-layer process. Ti and DLC thin films were subsequently deposited, and the resulting surfaces were characterised using reflected light optical microscopy and Raman spectroscopy to assess geometrical fidelity, coating conformity, and chemical–structural stability. Uniform coatings were successfully achieved on complex three-dimensional microtextures using both SLA systems. Substrates printed with the SLA-3500 exhibited well-defined layers and an average increase in valley curvature of approximately 2.8%, whereas Form 1 + printed samples showed a higher deviation of about 17.7% relative to the original design. Raman spectroscopy confirmed the presence of characteristic D and G bands at 1396 cm⁻¹ and 1589 cm⁻¹ in DLC-coated samples on both Accura^®^60 and Clear FLGPCL 02 substrates, indicating graphitic carbon domains while preserving the chemical integrity of the underlying polymer. Ti-coated surfaces exhibited increased broadband intensity between 1200 and 1420 cm⁻¹, attributed to resin–metal interactions. Despite minor variations in spectral intensity, no significant shifts in vibrational frequencies were observed, demonstrating comparable molecular stability of both substrate systems following coating deposition. These results establish a reliable framework for the fabrication and surface functionalization of architected polymer substrates, enabling future investigations into structure–property relationships and application-specific functional performance.

## Introduction

Additive manufacturing, commonly called three-dimensional (3D) printing or rapid prototyping, is a rapidly advancing technology that allows precise control over the architecture and dimensions to fabricate intrinsically complex shapes [1–3]; the ability to control and obtain desired dimensions with micron-level accuracy and precision can now be achieved using 3D printing [4, 5]. 3D printing coupled with sputtering deposition of single layer or multilayers can be employed to manufacture functional microstructures for use in aerospace, automotive, manufacturing, energy, transport, and biomedical fields [5–7].

A common additive manufacturing technique used for the manufacture of substrates is laser stereolithography (SLA), which is a 3D printing process that creates solid objects using a laser beam that photopolymerizes monomers, allowing greater control of the dimensions and features of the substrate compared to other additive manufacturing techniques [5, 8–10]; additionally, SLA provides the ability to manufacture an entire layer at once using a digital mask projection device that supersedes other 3D printing processes in yield.

The typical time to cure each layer is about 2 to 100 s, which results in a printing rate (height build) of about 5 mm/h to 1000 mm/h [9, 11, 12]; the total fabrication time depends on the size of the printed parts, desired resolution, layer thickness, and curing (or polymerization) kinetics. Since the SLA technique cures just one layer at a time, it can be used as a versatile printing method to create objects using various materials, including flexible and soft polymers [13, 14].

The current disadvantage of SLA technology is the limited number of available photoreactive polymers (epoxy resin, acrylic resin, polyurethane resin, polystyrene resin, dental resin), and these materials sometimes need to be biosafe; however, strenuous efforts and research in this direction have led to develop many photocurable polymers such as poly(ethylene glycol) diacrylate (PEGDA) [15–17], poly(2-hydroxyethyl methacrylate) (pHEMA) [15, 18], poly(ethylene glycol) dimethacrylate (PEGDMA) [15, 19, 20], and poly(propylene fumarate)/diethyl fumarate (PPF/DEF) [15, 21] that are better choices.

The robustness of SLA-fabricated microtextured substrates depends fundamentally on the integrity of both the polymer matrix and the coating-substrate interface. During stereolithographic processing, incomplete photopolymerization, anisotropic shrinkage and differential curing rates across layer boundaries generate residual tensile stresses that weaken inter-layer bonding and create microcracks within the polymer structure. These inherent flaws are exacerbated when thin-film coatings were subsequently deposited via magnetron sputtering, as the coating process introduces additional thermal stresses and may propagate existing defects through the polymer-coating interface. The resulting stress concentration sites can initiate brittle crack propagation under mechanical or thermal loading, compromising the long-term reliability of the component [22–26].

Therefore, the first approach involves characterisation of surface morphology through mechanical analysis, which can be subsequently complemented by assessment of layer adhesion quality and coating-substrate interfacial stability. This comprehensive evaluation is indispensable for optimizing SLA-fabricated coated systems and establishing design criteria for load-bearing applications [22–26]. Understanding the mechanisms of defect formation, crack propagation pathways, and coating-substrate interactions is therefore critical for developing SLA-fabricated coated systems suitable for demanding aerospace, biomedical, and precision engineering applications where failure is not tolerable.

This study highlights the protocol associated with the method and materials required to design and manufacture metamaterial-architecture-inspired coated 3D printed parts using a sequential fabrication process of SLA and direct current sputtering magnetron technique [5, 6, 10]. The research focuses on the influence of the SLA system and photo-resins employed, for which differences were observed while using an industrial and a low-cost printing system. In addition, surface functionalisation with different types of coatings was investigated. Here, the term “metamaterial-architecture-inspired” refers exclusively to the engineered surface geometry and does not imply the experimental demonstration of emergent metamaterial properties.

The coating materials were titanium (Ti) and diamond-like carbon (DLC), which were directed to the SLA-printed substrates using the DC magnetron sputtering technique [27–29]. In the process of doing so, the process parameters were also optimised and the influence of key parameters on the surface quality of surfaces on both uncoated surfaces was studied.

## Materials and methods

**Fig. 1:**
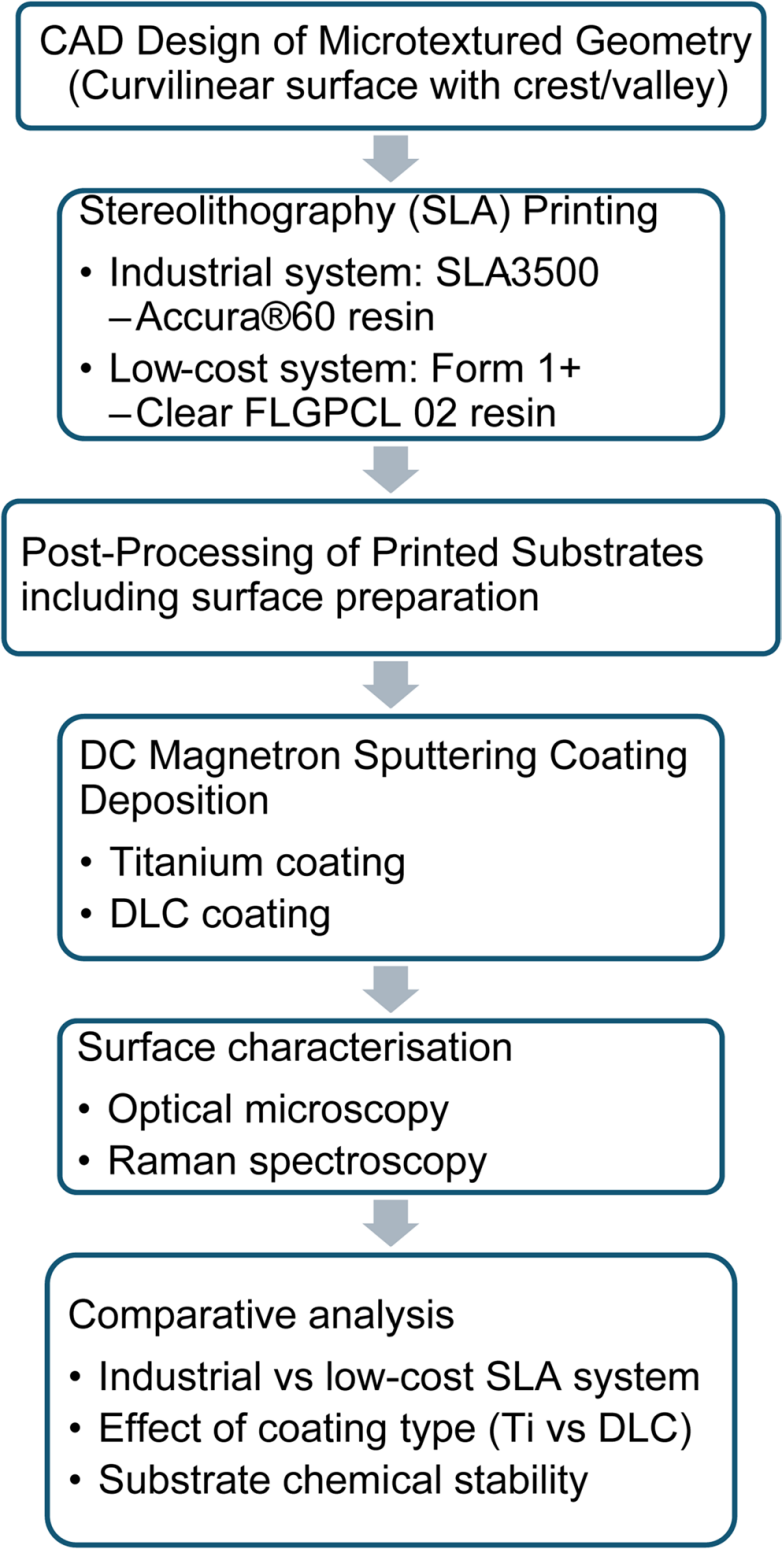
Experimental workflow for fabrication, coating, and characterisation of SLA-printed microtextured substrates

The experimental methodology in this work was sequentially developed. Figure 1 presents a flowchart summarizing the complete experimental workflow adopted in this study. All specimens were measured and analysed in triplicate.

### Preparation of polymer substrates

**Fig. 2:**
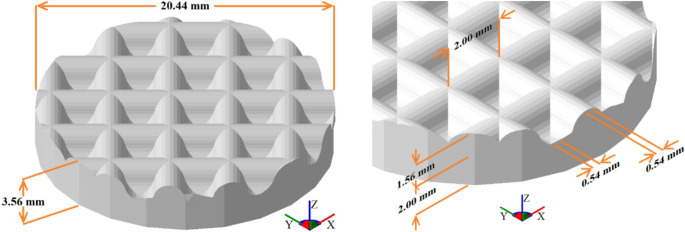
The substrate of the curvilinear section modeled using SketchUp software

The 3D printed substrates (see Fig. 2) were fabricated as circular disks measuring $\:20.44\:\mathrm{m}\mathrm{m}$ in diameter and $\:3.56\:\mathrm{m}\mathrm{m}$ in height, featuring a surface texture defined by a curved transversal section with a specific radius of curvature, designed using CAD software (SketchUp Make 2016, version 16.0.19911). Their production employed a commercial photosensitive resin via SLA with a layer thickness set to $\:0.1\:\mathrm{m}\mathrm{m}$. The geometry was selected to reflect typical features of rapid surface-coated prototypes widely employed in microfluidic applications such as lab-on-chip and organ-on-chip devices, where complex surface patterns including channels, grooves, ridges and textures spanning hundreds of microns to several millimeters are common.

Table 1 summarizes the material properties for substrates manufactured with Accura^®^60 resin (using the SLA-3500 printer, 3D Systems) and Clear FLGPCL 02 resin (using the Form 1 + printer, Formlabs) [5, 10, 30]. Both printers utilise a layer-by-layer fabrication approach with a resolution defined by the $\:100$-micron layer thickness, ensuring precise replication of intricate surface details [5, 10, 15, 30].

In the SLA method, a laser beam of UV light follows the path marked by the STL file as it scans across the surface in a vat with a photosensitive resin that polymerizes to form each layer of the substrate [5, 10, 30]. This method is fundamental to the history of additive manufacturing and provides one of the best compromises between part size, resolution and surface quality [9, 30–34].

**Table 1:** Properties of Accura^®^60 and clear FLGPCL 02 resins used in the printers SLA-3500 and form 1+

Parameter	Accura^®^60 resin	Clear FLGPCL 02 resin
	Value	Value
Density	$\:1.21\:\mathrm{g}/{\mathrm{c}\mathrm{m}}^{3}$	$\:1.10\:\mathrm{g}/{\mathrm{c}\mathrm{m}}^{3}$
Tensile strength	$\:58-68\:\mathrm{M}\mathrm{P}\mathrm{a}$	$\:61.5\:\mathrm{M}\mathrm{P}\mathrm{a}$
Young modulus	$\:2.7-3.1\:\mathrm{G}\mathrm{P}\mathrm{a}$	$\:2.7\:\mathrm{G}\mathrm{P}\mathrm{a}$
Elongation at break	$\:5-13\mathrm{\%}$	$\:5\mathrm{\%}$
Flexural modulus	$\:2.7-3.0\:\mathrm{G}\mathrm{P}\mathrm{a}$	$\:2.38\:\mathrm{G}\mathrm{P}\mathrm{a}$
Impact strength	$\:15-25\:\mathrm{J}/\mathrm{m}$	$\:29\:\:\mathrm{J}/\mathrm{m}$
Heat deflection temperature $\:(@66\mathrm{P}\mathrm{S}\mathrm{I})$	$\:{53-55\:}^{^\circ\:}\mathrm{C}$	$\:{78\:}^{^\circ\:}\mathrm{C}$

The detailed characterisation of substrate geometry and fabrication parameters is crucial for understanding how additive manufacturing affects surface topography and functionalization potential, providing a basis for optimizing coatings and subsequent device performance in targeted applications.

### Coatings fabricated by magnetron sputtering

In this study, direct current (DC) magnetron sputtering was employed to deposit Ti and DLC coatings, utilizing this technique’s inherent advantages for producing uniform, high-quality thin films on SLA-fabricated polymer substrates without thermal or chemical degradation of the underlying resin matrix; for Ti deposition, a rectangular pure titanium target ($\:200\times\:75\:{\mathrm{m}\mathrm{m}}^{2}$) with $\:99.9\mathrm{\%}$ purity was sputtered in an argon atmosphere at a working pressure of $\:4\times\:{10}^{-3}\:\mathrm{m}\mathrm{b}\mathrm{a}\mathrm{r}$ with a DC magnetron power of $\:\:200\:\mathrm{W}$. The process lasted approximately $\:7$ minutes, resulting in a Ti film about $\:200\:\mathrm{n}\mathrm{m}$ thick.

In the case of DLC coatings, a pure graphite target ($\:400\times\:100\:{\mathrm{m}\mathrm{m}}^{2}$) with $\:99.9\mathrm{\%}$ purity was utilized. The magnetron operated in pulsed-DC mode at $\:150\:\mathrm{k}\mathrm{H}\mathrm{z}$ frequency and $\:4\:{\upmu\:}\mathrm{s}$ pulse duration. The purity of the sputter targets was $\:99.0\%.\:$The base pressure of the system was 5 × 10^− 6^ mbar and argon working pressure was maintained at 7 × 10^− 3^ mbar. Substrates were mounted on a parallel rotation stage positioned 8 cm from the target during deposition. After $\:90$ minutes, a DLC layer of approximately 40 nm thickness was obtained. The film thicknesses for both Ti and DLC coatings were measured using a profilometer on masked glass slides.

The sputtering process enables precise control over coating thickness and uniformity, essential for maintaining surface texture fidelity and functional properties; such detailed deposition characterisation directly informs the understanding of coating performance, adhesion, and the resulting modification of the substrate’s surface chemistry and topography, which are critical for tailoring materials in advanced applications.

### Optical microscopy

Images of the textured surfaces were acquired through optical microscopy with a lens of $\:10\mathrm{X}$ on the Olympus DSX500 microscope; the raw images were processed using DSX-BSW 2.1.4 software (Olympus Corporation). The measurements of the surfaces, coated and uncoated, reconstructed digitally were obtained with the motorized focus to a resolution of $\:0.01\:{\upmu\:}\mathrm{m}$ and accuracy of $\:\pm\:3\mathrm{\%}$. This method aid to quantify, validate and guarantee the quality of the external surfaces of any object manufactured by SLA. Surface roughness characterisation was performed by analyzing three-dimensional topography profiles extracted from reflected light optical microscopy images. The average surface roughness along the z-axis was measured at five distinct locations per specimen using image analysis software.

### Raman spectroscopy

The characterisation by Raman spectroscopy was performed in-situ to determine the vibrations and bands of present species on the surfaces of the substrates using a Horiba Scientific confocal spectrometer (LabRam HR, HORIBA); this equipment comes equipped with a laser of $\:532\:\mathrm{n}\mathrm{m}$ and a microscope objective of $\:100\mathrm{X}$. The Raman measurements were obtained directly on samples with a laser power of $\:15\:\mathrm{m}\mathrm{W}$ and $\:20\:\mathrm{m}\mathrm{W}$ based on the material’s natural (free) vibrational modes.

The settings were established to obtain spectra with the highest signal-to-noise relationship according to the chemical nature of the materials used in this research, considering epoxy photoreactive resins and polymer with two coating types. Notwithstanding, in some samples, the fluorescence was considerable, and the signal of the noise of the spectrum did not allow identification of the bands corresponding to the material. Hence, the power of the laser was modified to reduce the noise signal to achieve an accurate definition of the vibration modes. LabSpec 6 software (Horiba Scientific) was used for Raman signal acquisition and analysis. Each sample was scanned in the range of $\:25\:{\mathrm{c}\mathrm{m}}^{-1}$ to $\:400\:{\mathrm{c}\mathrm{m}}^{-1}$. The mean values and standard deviations were analysed.

## Results and discussions

This research analyses two distinct regions, the crest and valley (these terms describe periodic geometric features), of textures fabricated using two different 3D printing systems, each employing specific resin types (Accura^®^ฏ60 and Clear FLGPCL 02) and coated with titanium (Ti) and diamond-like carbon (DLC) films via magnetron sputtering; optical microscopy serves as a primary tool for assessing the geometric fidelity of the printed components from both printers, while Raman spectroscopy complements this by identifying chemical species deposited, particularly within textured recesses. Together, the combined morphological and chemical analyses provide a comprehensive characterisation framework that verifies dimensional precision and coating uniformity while elucidating surface chemistry modifications, which are crucial for optimizing performance in engineering and biomedical applications.

### General issues

**Fig. 3:**
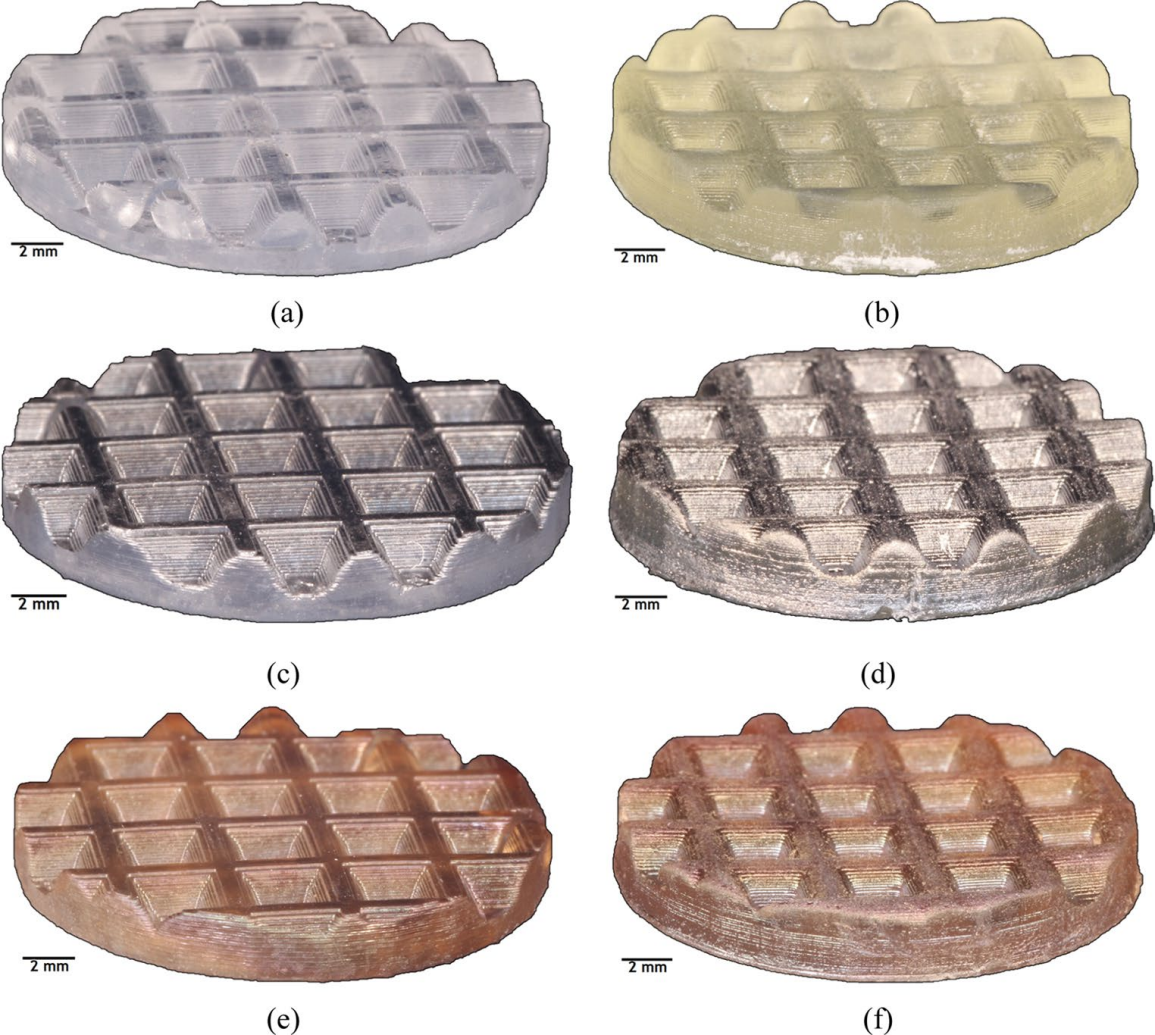
Specimens with surface textures including a network of $\:1\:mm$-diameter crossing ribs; (**a**) and (**b**) are the uncoated samples (as printed) using SLA-3500 and Form 1+, respectively, (**c**) Ti-coated samples printed by SLA-3500, (**d**) Ti-coated samples printed by Form 1+, (**e**) DLC-coated samples printed by SLA-3500, (**f**) DLC-coated samples printed by Form 1+

The substrates manufactured by the SLA technique in the SLA-3500 and Form 1 + printers, with photoreactive resins Accura^®^60 and Clear FLGPCL 02, respectively, are presented in Fig. 3(a) and Fig. 3(b). Figure 3(c), and Fig. 3(d) are the images of the Ti-coated substrates and Fig. 3(e) and Fig. 3(f) are the DLC coated samples.

The observed surface irregularities in samples fabricated using the Form 1 + system arise from the lower optical resolution and reduced laser spot control compared to the industrial SLA-3500 system. In SLA processes, laser beam stability and resin photopolymerization kinetics strongly influence layer bonding and surface smoothness, particularly in curved geometries. Similar printer-dependent surface deviations have been reported in previous studies comparing industrial and desktop SLA platforms, where reduced energy density leads to diminished interlayer fusion and increased surface waviness.

Table 2 compares the geometric measurements of printed substrates against their original CAD designs, revealing that parts produced by the Form 1 + printer demonstrate accuracy more closely aligned with design specifications. However, both printers yield a surface finish acceptable relative to one another. It is critical to consider the tolerance values inherent to additive manufacturing processes based on equipment capabilities and intended application. In this study, a tolerance margin of approximately $\:\pm\:\:50\:{\upmu\:}\mathrm{m}$ was deemed appropriate.

**Table 2:** Values of the length, mass, and volume measurements of the substrates manufactured in the printers SLA-3500 and form 1+

Substrate type	CAD model dimensions				Experimental measurement of 3D parts			
	Radius (cm)	Thickness (cm)	Volume (cm^3^)	Mass* (g)	Radius (cm)	Thickness (cm)	Mass (g)	Volume* (cm^3^)
SLA-3500	$$\:1.022$$	$$\:0.356$$	$$\:1.150$$	$$\:1.392$$	$$\:1.019$$	$$\:{0.285}^{**}$$	$$\:0.880$$	$$\:0.727$$
Form 1+	$\:1.022$	$\:0.356$	$\:1.150$	$\:1.265$	$\:1.019$	$\:0.335$	$\:0.976$	$\:0.887$

* Calculated value having as reference the value of density reported in Table 1

** This measurement is smaller because the surface of the device was polished to remove the support structures

On the other hand, the manufacturing process performed by the SLA industrial system presents a particular issue; generally, the objects were printed with support that hold them to the platform. Therefore, to have a product with a good finish, the surface in contact with the support must eventually be polished; unlike SLA, this limitation does not occur with the Form 1 + low-cost system because it can be printed on the platform without support depending on the substrate type.

### Microscopic characterisation

**Fig. 4:**
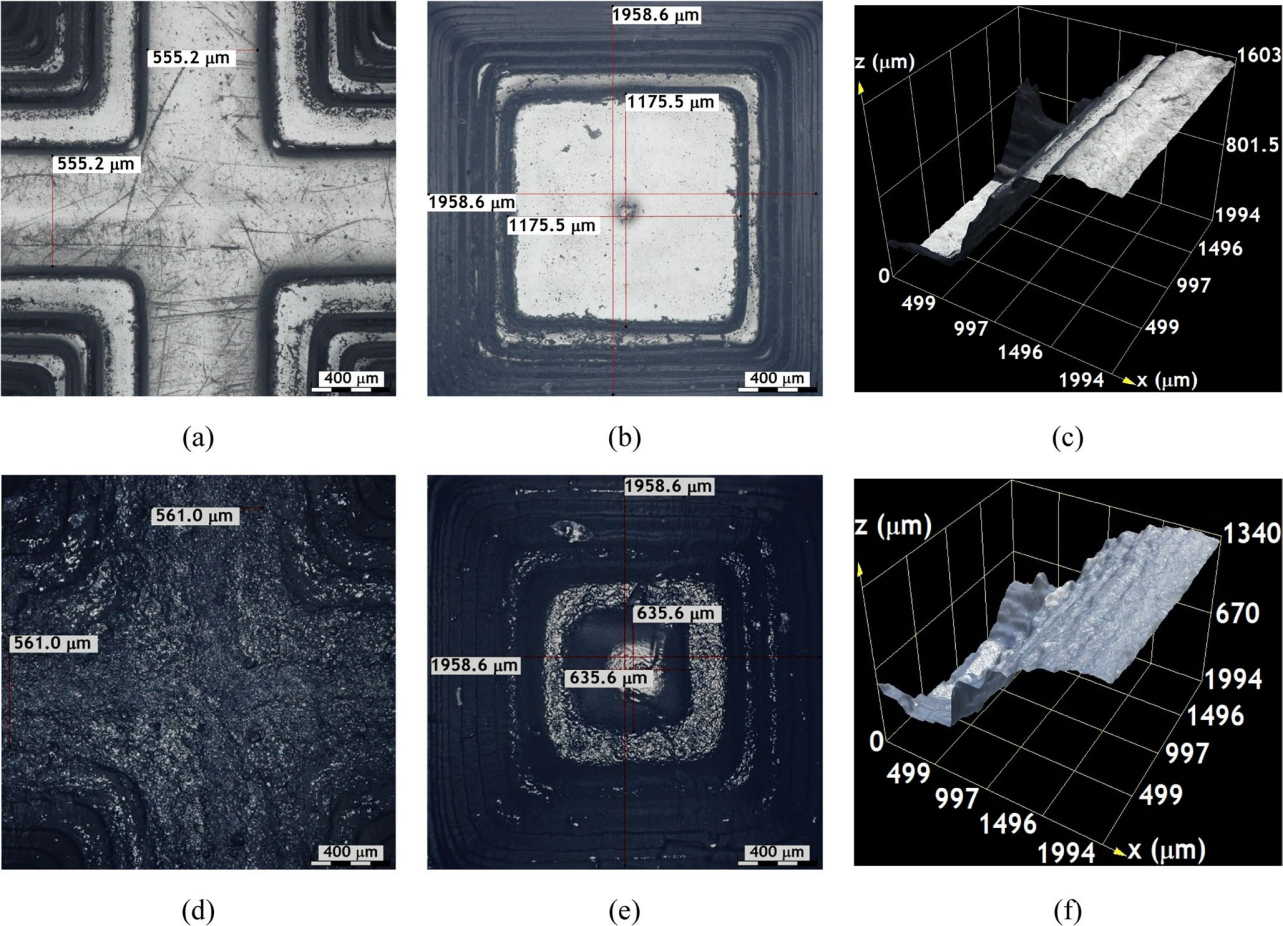
Images captured using $\:10\mathrm{X}$ objective lenses on uncoated textured surfaces; (**a**), (**b**), and (**c**) correspond to the crest, valley, and the height between crest and valley of the substrates printed by the SLA-3500 printer, respectively. Similarly, (**d**), (**e**), and (**f**) show the crest, valley and corresponding height measurements of substrates manufactured with the Form 1 + printer

**Fig. 5:**
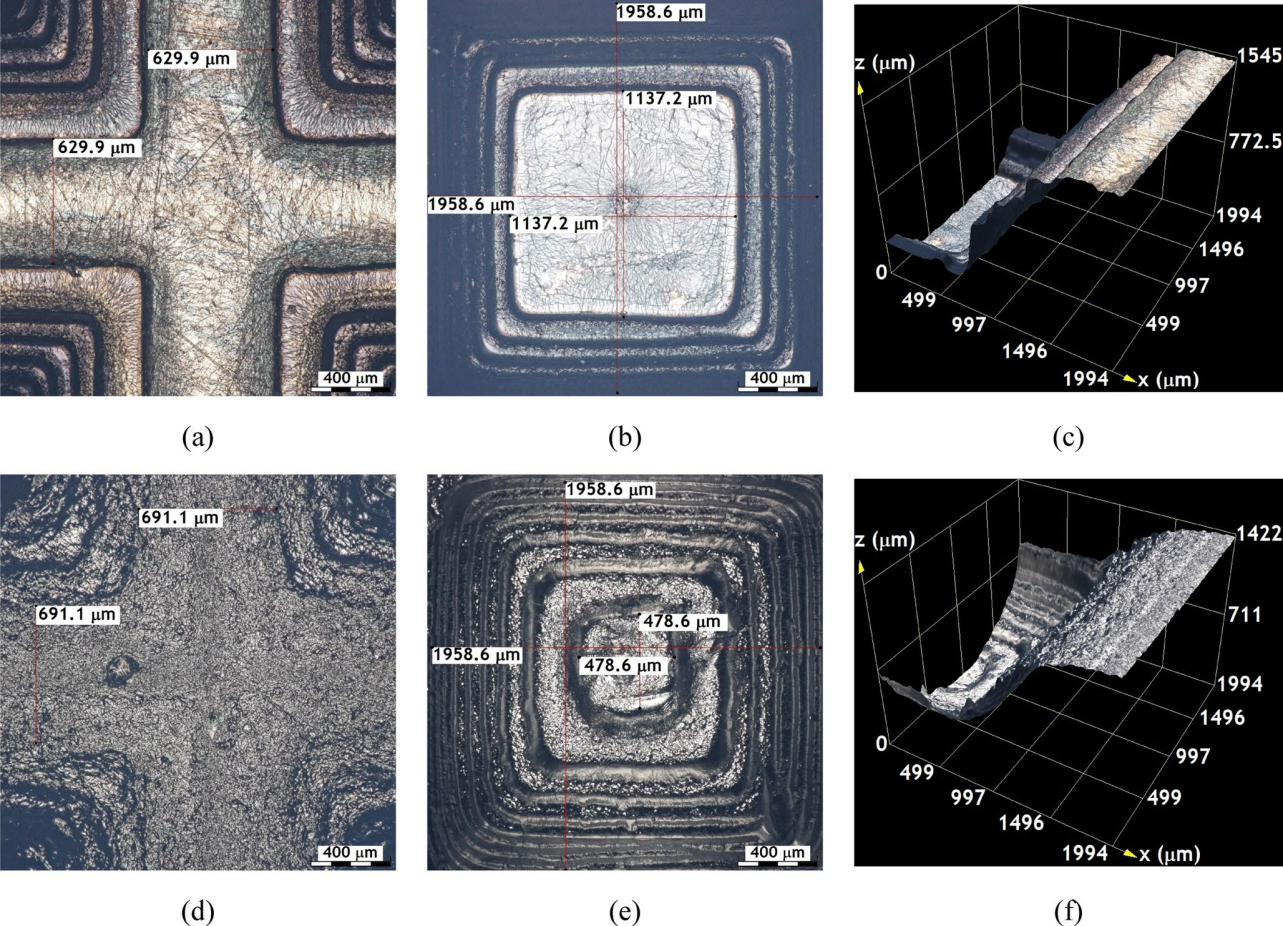
Images captured using $\:10\mathrm{X}$ objective lenses on the Ti-coated textured surfaces; (**a**), (**b**), and (**c**) correspond to the crest, valley, and the height between crest and valley of the substrates printed by the SLA-3500 printer, respectively. Similarly, (**d**), (**e**), and (**f**) show the crest, valley, and corresponding height measurements of substrates manufactured with the Form 1 + printer

**Fig. 6:**
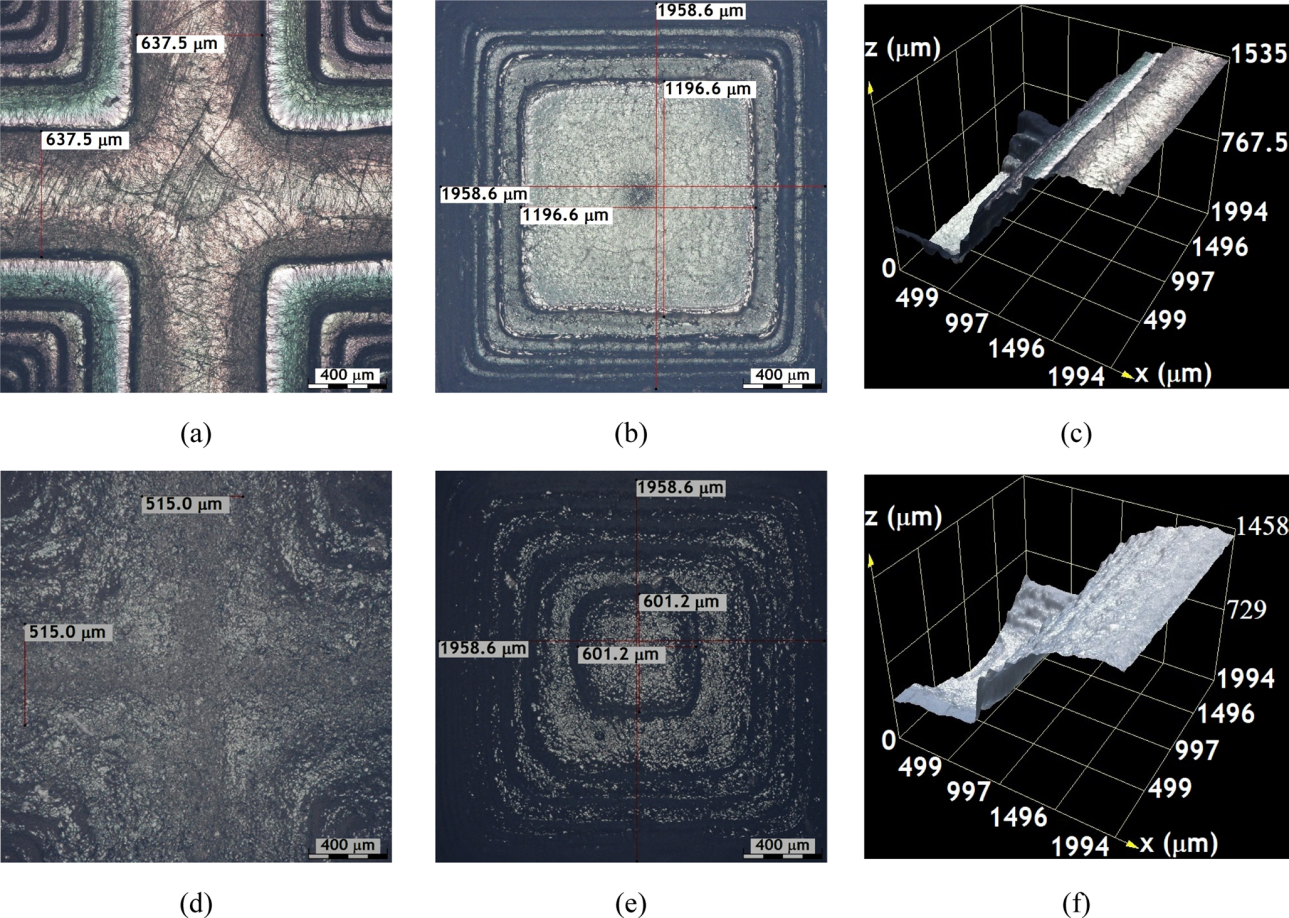
Images captured using $\:10\mathrm{X}$ objective lenses on the DLC-coated textured surfaces; (**a**), (**b**), and (**c**) correspond to the crest, valley, and the height between crest and valley of the substrates printed by the SLA-3500 printer, respectively. Similarly, (**d**), (**e**), and (**f**) show the crest, valley, and corresponding height measurements of substrates manufactured with the Form 1 + printer

Figures 4 and 5, and Fig. 6 provide detailed optical microscopy images of surface features on samples printed with the SLA-3500 using Accura^®^60 resin and the Form 1 + with Clear FLGPCL 02 resin. These include uncoated samples as well as those coated with approximately $\:200\:\mathrm{n}\mathrm{m}$ of Ti and $\:40\:\mathrm{n}\mathrm{m}$ of DLC, exhibiting uniform coating thickness around the entire cross sections.

The average roughness measured along the *z*-axis of the 3D printed textures reveals the layer-by-layer manufacturing process; however, the final geometry remains largely unaffected, as the designed features are roughly twice the machine’s resolution capability. From a surface characterisation standpoint, these microscopic observations confirm the capability to fabricate intricate 3D microtextures with coatings applied uniformly and without detrimental distortions. This is critical for tailoring surface morphology and properties through additive manufacturing and physical vapor deposition techniques, which underpin a wide range of applications demanding precise structural and chemical surface control.

In Fig. 4, the texture of the substrates manufactured with the SLA-3500 printer can be seen to have a better surface finish with well-defined contours compared to those printed on Form 1+, which is in agreement with the results shown in Fig. 3.

The value of the thickness of the last layer of the crest manufactured by the SLA-3500 printer was approximately 555.2 microns (see Fig. 4(a)), while the one manufactured by Form 1 + printer was about $\:561$ microns (see Fig. 4(d)).

The improved surface finish and sharper contour definition observed in substrates fabricated using the SLA-3500 system can be attributed to its higher laser power stability and finer scanning control, which promote more uniform photopolymerization across each layer. This enhanced control reduces staircase effects and curvature distortion, particularly in microtextured features. Previous investigations have demonstrated that industrial SLA systems exhibit superior dimensional accuracy and curvature fidelity when compared to low-cost printers, especially for features approaching the machine resolution limit. Figure 4(b) and Fig. 4(e) present the deepest layers of the valleys of the surface textures manufactured with the SLA-3500 and Form 1 + printers, respectively.

The internal layering of the SLA-3500 printed parts displays well-defined layers but with lower precision regarding curvature control, as evidenced by an approximate $\:1175.5\:{\upmu\:}\mathrm{m}$ distance measured between the deepest layer boundaries. Conversely, the Form 1 + prints show a shorter length of about $\:635.6\:{\upmu\:}\mathrm{m}$, indicative of superior performance in fabricating curved geometries. Surface topography analyses between crests and valleys show an average crest-valley distance of $\:1496.8\:{\upmu\:}\mathrm{m}$ for Accura^®^60 samples via SLA-3500, compared to $\:1283.3\:{\upmu\:}\mathrm{m}$ for Clear FLGPCL 02 samples produced by the Form 1+ (Fig. 4(c) and Fig. 4(f)). This suggests that the SLA-3500 achieves higher accuracy in layer replication than the Form 1+.

Regarding curvature, surfaces printed with the SLA-3500 demonstrate increases of approximately $\:2.8\mathrm{\%}$ at the valleys and $\:117.7\mathrm{\%}$ at the crests relative to the designs (see Fig. 4(a) and Fig. 4(b)), whereas the Form 1 + prints show increases around $\:3.9\mathrm{\%}$ at the crests and $\:17.7\mathrm{\%}$ at the valleys (Fig. 4(d) and Fig. 4(e)). Overall, while the SLA-3500 yields more precise crest curvatures and clearer contour definition with better surface finish, Form 1 + tends to better reproduce valley curvatures.

The increased curvature observed at valley regions in SLA-3500-printed substrates is likely caused by cumulative resin shrinkage during successive layer curing, which is amplified in concave geometries where light scattering and overcuring occur. Conversely, the Form 1 + system exhibits improved valley reproduction, potentially due to lower energy exposure per layer, which reduces over-polymerization effects. Similar curvature-dependent dimensional deviations in SLA-fabricated components have been widely reported.

Figure 5 illustrates the optical micrographs of Ti-coated substrates, highlighting that the surface integrity and sharpness of contours remain intact post-coating, which assures measurement reliability. Images of crests (Fig. 5(a) and Fig. 5(d)), valleys (Fig. 5(b) and Fig. 5(e)), and digitally reconstructed surfaces between them (Fig. 5(c) and Fig. 5(f)) reveal that the original CAD-defined geometries are well preserved after titanium deposition. Besides functional and chemical advantages, the Ti coating confers appealing aesthetic qualities of interest for industrial decorative applications.

The DLC coated surface textures shown in Fig. 6 are analogous to the Ti coated samples, as the crests shown in Fig. 6(a) and Fig. 6(d), the valleys shown in Fig. 6(b) and Fig. 6(e), and the distances between them shown in Fig. 6(c) and Fig. 6(f) for textures fabricated using the SLA 3500 and Form 1 plus printers exhibit uniform coloration with no evidence of coating delamination. The coatings adhere well and exhibit no immediate peeling, significantly improving the surface appearance compared to uncoated substrates [33, 34]; moreover, the overall geometry remains unchanged after coating, with layer thicknesses ranging from approximately $\:40$ to $\:200$ nanometers.

Optical microscopy analyses shown in Figs. 4 and 5, and Fig. 6 demonstrate that combining stereolithography (SLA) with magnetron sputtering produces micron-scale substrates featuring complex 3D geometries or textured surfaces that can be effectively functionalized; these textured surfaces, including perforations up to $\:500$ microns in diameter, successfully incorporate different coating types (Ti and DLC), confirming the potential for versatile surface modifications of SLA-fabricated photosensitive resin microdevices.

### Spectroscopic characterisation

**Fig. 7:**
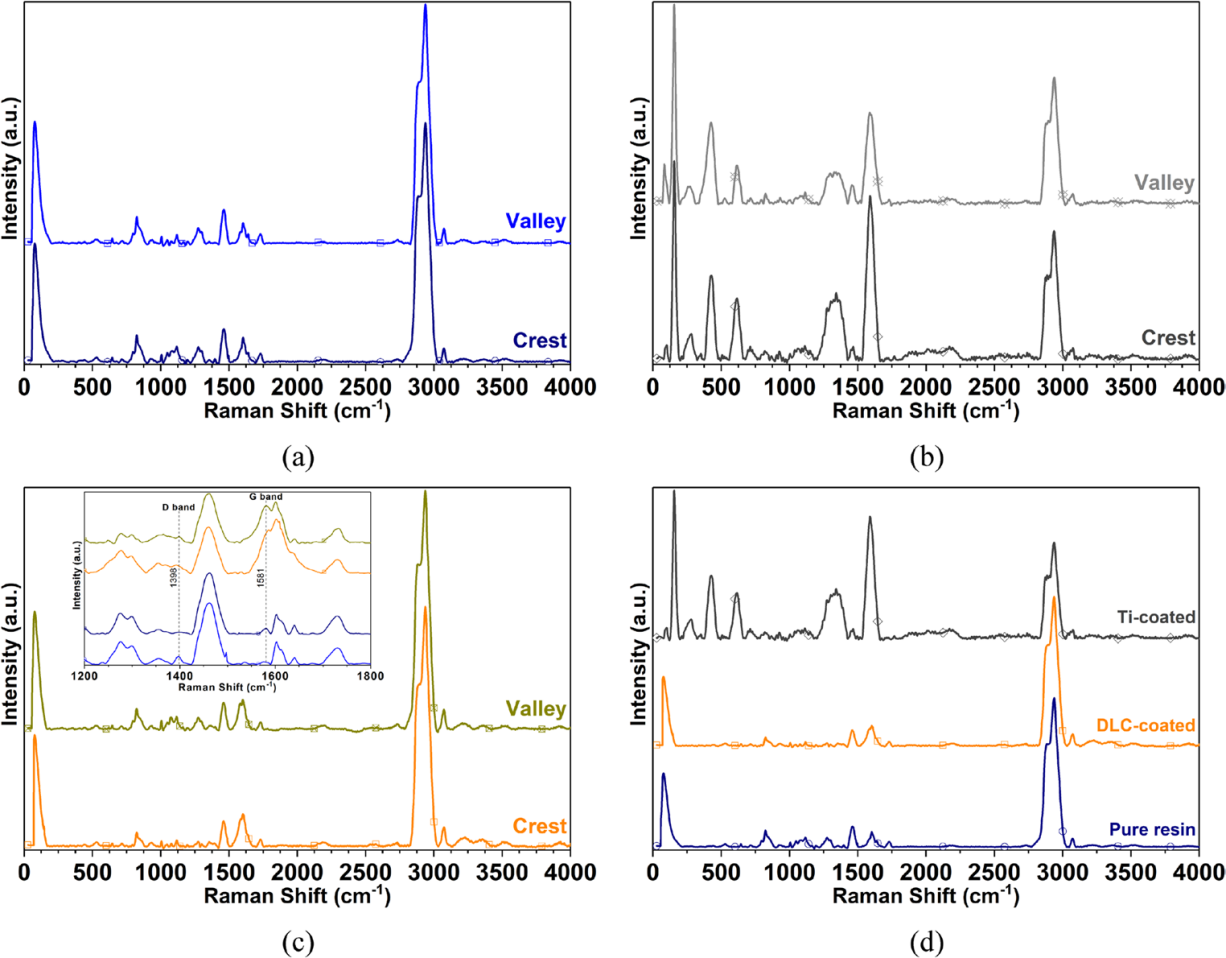
Raman spectra acquired on the crests and valleys of the specimen printed in the SLA-3500 with Accura^®^60 resin. (**a**) uncoated surfaces, (**b**) Ti-coated, (**c**) DLC-coated, and (**d**) spectral comparison on the crest

**Fig. 8:**
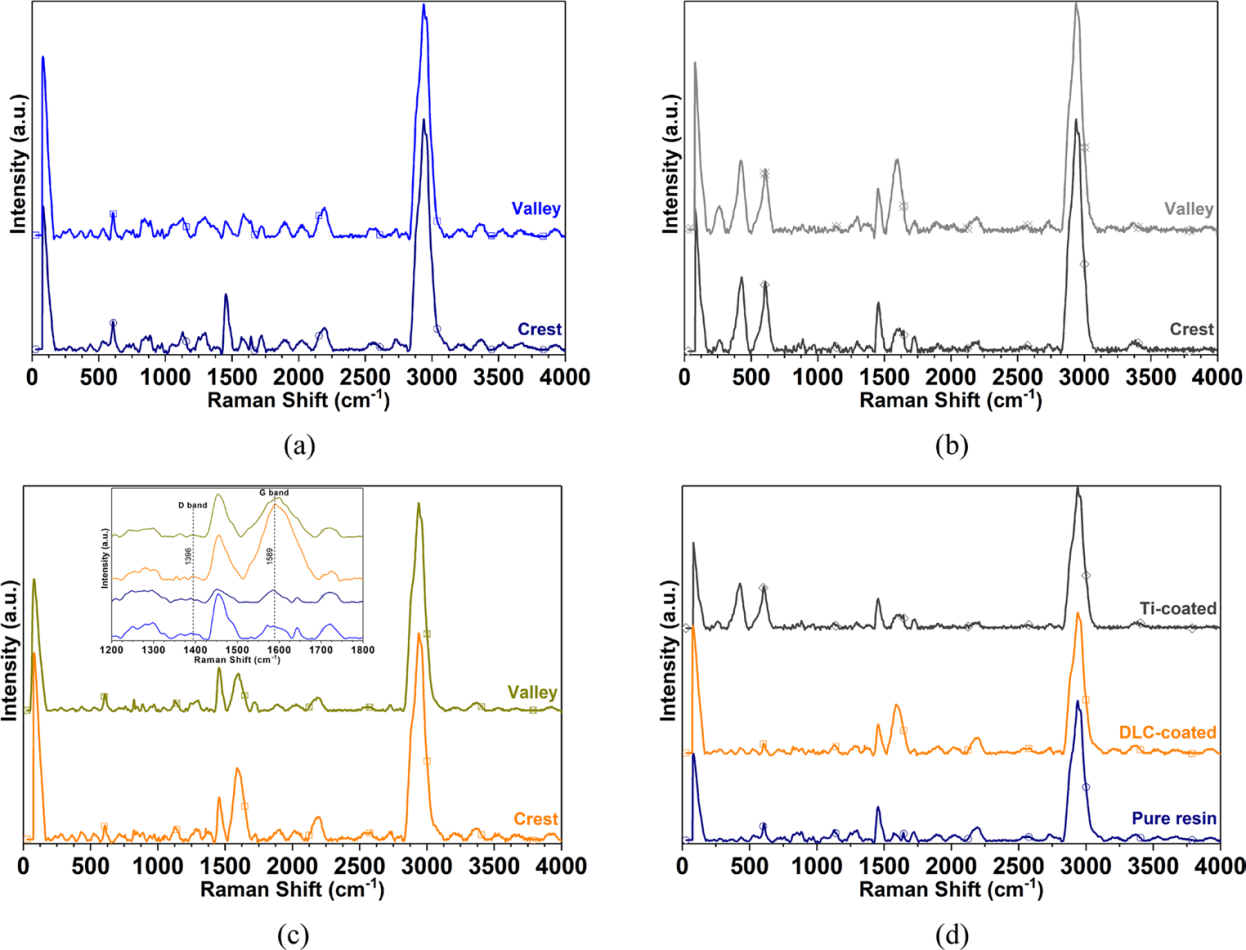
Raman spectra acquired on the crests and valleys of the substrate printed in Form 1 + with Clear FLGPCL 02 resin. (**a**) uncoated surfaces, (**b**) Ti-coated, (**c**) DLC-coated, and (**d**) spectral comparison on the crest

Micro-Raman spectroscopy was applied to both uncoated and coated photosensitive resin substrates (modified with Ti and DLC), and the measurements acquired in spectral mode are shown in Figs. 7 and 8. Unlike reflection mode, spectral mode interrogates a defined volume extending from the surface into the material, typically between $\:1$ and $\:10$ microns, depending on both the material’s properties and instrumental parameters. This feature makes it particularly valuable for exploring both surface and near-surface regions, revealing compositional gradients or modifications due to coating processes.

The micro-Raman approach thus enables in situ, non-destructive identification of chemical bonds and structural motifs at the surface and subsurface, directly connecting spectroscopic features to coating uniformity, chemical compatibility, and the preservation or modification of functional groups; such analysis is a cornerstone in the elucidation and tailoring of surface-engineered materials, supporting design decisions in fields ranging from advanced coatings to biomedical devices and optoelectronic components. The spectra shown in Fig. 7 on the valley and crest of the substrate show bands corresponding to the thermo-stable polymers associated with the epoxy groups of the constituent resin of the substrate; the epoxide vibrations were in the range of $\:{1200\:\mathrm{c}\mathrm{m}}^{-1}$ and $\:{1480\:\mathrm{c}\mathrm{m}}^{-1}$. Moreover, between $\:{1600\:\mathrm{c}\mathrm{m}}^{-1}$ and $\:{1700\:\mathrm{c}\mathrm{m}}^{-1}$, there were aromatic ring chain vibrations; the vibrations between $\:1460\:{\mathrm{c}\mathrm{m}}^{-1}$ and $\:{1465\:\mathrm{c}\mathrm{m}}^{-1}$ can be attributed to the bending modes $\:{\mathrm{C}\mathrm{H}}_{\mathrm{2,3}}$ [35, 36].

Figure 7 presents Raman spectra of specimens manufactured using Accura^®^60 resin in the uncoated state and after coating with titanium and diamond-like carbon coatings. Characterisation of the uncoated Accura^®^60 substrate shown in Fig. 7(a) enabled the establishment of baseline spectroscopic features corresponding to the chemical fingerprint of the unmodified polymer. Three prominent Raman peaks were observed at 1455 cm^− 1^ with medium intensity, 2883 cm^− 1^ with strong intensity, and 2935 cm^− 1^ with very strong intensity, which are characteristic of C-H bond vibrations. Additional medium intensity peaks observed at 822 cm^− 1^ and 1600 cm^− 1^ can be attributed to C-C and C = O bond vibrations, respectively. The bands at $\:{1600\:\mathrm{c}\mathrm{m}}^{-1}$ to $\:{2000\:\mathrm{c}\mathrm{m}}^{-1}$ were assigned to the $\:\mathrm{C}=\mathrm{O}$ bond vibrations, between $\:{500\:\mathrm{c}\mathrm{m}}^{-1}$ to $\:{800\:\mathrm{c}\mathrm{m}}^{-1}$ to the $\:\mathrm{C}-\mathrm{O}-\mathrm{C}$ bond symmetric stretching vibrations and $\:\mathrm{C}-\mathrm{O}$ bond vibrations, and the region between $\:{1000\:\mathrm{c}\mathrm{m}}^{-1}$ and $\:{400\:\mathrm{c}\mathrm{m}}^{-1}$ correspond to the $\:\mathrm{C}-\mathrm{O}-\mathrm{C}$ bond asymmetric stretching vibrations and to the $\:\mathrm{C}{\mathrm{H}}_{2}$ bond vibrations [37].

The Raman spectrum acquired on Ti-coated surfaces (crest and valley, Fig. 7(b)) revealed peaks at $\:{157\:\mathrm{c}\mathrm{m}}^{-1}$. These can be attributed to brookite whilst the peaks at $\:{427\:\mathrm{c}\mathrm{m}}^{-1}$ and $\:{614\:\mathrm{c}\mathrm{m}}^{-1}$ are that of rutile $\:\mathrm{a}\mathrm{n}\mathrm{d}\:\mathrm{t}\mathrm{h}\mathrm{e}\:$bands between $\:{219\:\mathrm{c}\mathrm{m}}^{-1}$ and $\:{324\:\mathrm{c}\mathrm{m}}^{-1}$ can be assigned to the multiphoton process [38–44] which occurs due to oxidation of the coating of Ti produced by the Accura^®^60 resin chemical composition. This oxidation does not occur during sputtering, but it is due to the subsequent exposure to ambient conditions, as all the specimens were deposited simultaneously and have a metallic color due to the Ti coating; the other representative peaks correspond to the vibration modes determined in the uncoated surface (see Fig. 7(a)).

In surface DLC-coated (Fig. 7(c)), spectra collected at both crest and valley positions reveal that most vibrational features of the original resin are retained, but with new signals at $\:{1396\:\mathrm{c}\mathrm{m}}^{-1}$ and $\:{1589\:\mathrm{c}\mathrm{m}}^{-1}\:$assigned to D ($\:{\mathrm{A}}_{1\mathrm{g}}$) and G ($\:{\mathrm{E}}_{2\mathrm{g}}$) bands indicated the presence of graphitic carbon domains. These additional Raman signatures evidence chemical and structural changes introduced by the DLC layer, while the persistence of the main polymeric peaks signals that the underlying substrate remains chemically stable [29, 45–47].

The pure epoxy resin main peaks were at $\:{1460\:\mathrm{c}\mathrm{m}}^{-1}$ and $\:{1600\:\mathrm{c}\mathrm{m}}^{-1}$; although the surface was DLC-coated, the Raman spectra does not show significant differences. Nevertheless, it is possible to find clear differences between coated surfaces and uncoated surfaces by selective zooming in the range of $\:{1200\:\mathrm{c}\mathrm{m}}^{-1}$ and $\:{1800\:\mathrm{c}\mathrm{m}}^{-1}$.

To summarize, four main peaks were detected in the DLC-coated epoxy and they were at $\:1398\:{\mathrm{c}\mathrm{m}}^{-1}$, $\:1461\:{\mathrm{c}\mathrm{m}}^{-1}$, $\:1581\:{\mathrm{c}\mathrm{m}}^{-1}$ and $\:1602\:{\mathrm{c}\mathrm{m}}^{-1}$. As in the neat epoxy case, no shift in the peaks was detected.

Accordingly, a comparison between the spectrum of the uncoated surfaces with the Ti and DLC-coated surfaces (see Fig. 7(d)), a broadband between $\:1200\:{\mathrm{c}\mathrm{m}}^{-1}-1420\:{\mathrm{c}\mathrm{m}}^{-1}$ in the spectrum acquired on the Ti-coated surface (Fig. 7(b) and Fig. 7(d)) shows that the band increases its intensity. This phenomenon is probably associated with the interaction between the resin and Ti, where an overlap occurs with the vibration mode associated with this element at $\:1370\:{\mathrm{c}\mathrm{m}}^{-1}$ [28, 35, 48, 49].

On the other hand, in the spectrum of DLC-coated surfaces, the carbon-related peaks are more defined and intense, due to the intramolecular forces of the vibrational excitations of carbon present in the DLC coating; consequently, it has an amorphous structure with sensitivity to the *sp*^*2*^ and *sp*^*3*^ bonding of the carbon atoms and the nanocrystalline clusters, with short-range and medium-range order of *sp*^*2*^ and *sp*^*3*^ [50–52].

Figure 8 shows the Raman spectra collected from substrates fabricated with Clear FLGPCL 02 resin, both uncoated and after deposition of Ti and DLC layers. In Fig. 8(a), three prominent peaks were identified at $\:{1455\:\mathrm{c}\mathrm{m}}^{-1}$, $\:{2940\:\mathrm{c}\mathrm{m}}^{-1}$, and $\:{2960\:\mathrm{c}\mathrm{m}}^{-1}$, corresponding to the characteristic $\:\mathrm{C}-\mathrm{H}$ bond vibrations in the pristine resin. A faint signal at $\:{600\:\mathrm{c}\mathrm{m}}^{-1}$ is linked to $\:\mathrm{C}-\mathrm{C}$ bond vibration, while the spectral region between $\:{800\:\mathrm{c}\mathrm{m}}^{-1}$ and $\:{950\:\mathrm{c}\mathrm{m}}^{-1}$ is indicative of $\:\mathrm{C}-\mathrm{O}-\mathrm{C}$ stretching modes. Further, the interval from $\:{1000\:\mathrm{c}\mathrm{m}}^{-1}$ to $\:{1150\:\mathrm{c}\mathrm{m}}^{-1}$ reflects asymmetric $\:\mathrm{C}-\mathrm{O}-\mathrm{C}$ stretching, the peak at $\:{1408\:\mathrm{c}\mathrm{m}}^{-1}$ can be ascribed to asymmetric $\:{\mathrm{C}\mathrm{H}}_{3}$ bond stretching, and the signal at $\:1710\:{\mathrm{c}\mathrm{m}}^{-1}$ was attributed to the $\:\mathrm{C}=\mathrm{O}$ bond vibration. Additional minor bands in the $\:{2700\:\mathrm{c}\mathrm{m}}^{-1}$ – $\:{2850\:\mathrm{c}\mathrm{m}}^{-1}$ and $\:{3150\:\mathrm{c}\mathrm{m}}^{-1}$ – $\:{4000\:\mathrm{c}\mathrm{m}}^{-1}$ regions were related to $\:\mathrm{O}-{\mathrm{C}\mathrm{H}}_{3}$ and $\:-\mathrm{H}$ bond vibrations, respectively [10].

The analysis in Fig. 8(b) shows that, once the resin surface is coated with Ti, new spectral features emerge, including bands at $\:426\:{\mathrm{c}\mathrm{m}}^{-1}$ and $\:605\:{\mathrm{c}\mathrm{m}}^{-1}$, which are indicative of rutile phases formed on the surface [38–44]. Additionally, broad bands between $\:229\:{\mathrm{c}\mathrm{m}}^{-1}$ and $\:293\:{\mathrm{c}\mathrm{m}}^{-1}$ correspond to multiphoton vibrational modes, while other relevant peaks are consistent with vibrational characteristics of the base resin (see Fig. 8a). The comparable spectral profiles between measurements taken at the crest and valley regions demonstrate uniform titanium coverage across the textured substrate, essential for achieving functional surface modifications.

Figure 8(c) shows the Raman response of the same resin after DLC deposition, with spectra collected at both crest and valley positions. The principal peaks resemble those in the uncoated material, however, the emergence of strong signals at $\:1396\:{\mathrm{c}\mathrm{m}}^{-1}$ and $\:1589\:{\mathrm{c}\mathrm{m}}^{-1}$ denotes the presence of D ($\:{\mathrm{A}}_{1\mathrm{g}}$) and G ($\:{\mathrm{E}}_{2\mathrm{g}}$) bands associated with graphitic carbon, confirming the effective integration of DLC and its structural characteristics relative to the Ti-coated and unmodified surfaces (see Fig. 8(d)) [28, 45–47, 50–52].

Notably, despite minor differences in spectral intensity, neither the chemical composition nor the vibrational frequencies associated with $\:\mathrm{C}$-based bonds differ significantly between Accura^®^60 and Clear FLGPCL 02 substrates, indicating that the main molecular structure remains stable through subsequent surface modifications (see Fig. 8(d)).

These Raman signatures provide a clear molecular fingerprint of the surfaces at each processing stage, revealing both chemical and structural changes at high spatial resolution. This is particularly critical for the rational design of materials in advanced engineering applications, where surface chemistry and uniformity dictate functional properties such as wettability, adhesion, and compatibility with other coatings. Ultimately, these findings underscore the relevance of additive manufacturing combined with surface analysis, confirming that complex resin substrates can be reproducibly coated to achieve tailored functionality suitable for high-performance devices in material science.

## Conclusions

This study demonstrates a novel methodology for successful integration of laser stereolithography and direct current magnetron sputtering for the fabrication of three-dimensional polymer substrates featuring metamaterial-like-architecture coated with functional titanium and diamond like carbon thin films. Two stereolithography systems were investigated, namely the industrial SLA 3500 printer using Accura^®^60 resin and the low-cost Form 1 + printer using Clear FLGPCL 02 resin, to evaluate how printing resolution and process stability influence geometric fidelity and coating conformity on substrates with metamaterial-like architecture and complex curved geometries.

Optical microscopy confirmed that both stereolithography systems enabled the deposition of continuous and well adhered titanium and diamond-like carbon coatings across intricate features associated with metamaterial-like architecture, including micron scale crests, valleys and perforated regions, without evidence of delamination or geometric distortion. Quantitative analysis revealed that substrates fabricated using the SLA 3500 system exhibited significantly higher geometric accuracy, with an average curvature deviation of approximately 2.8% relative to the original design, whereas substrates produced using the Form 1 + system showed a higher deviation of approximately 17.7%. Despite these differences in geometric precision, both printing platforms achieved comparable coating uniformity, demonstrating that conformal thin film deposition can be reliably achieved on metamaterial-like architectures even when using cost effective stereolithography systems.

Raman spectroscopic analysis provided detailed insight into coating substrate interactions and chemical stability in metamaterial-like architecture-based substrates. Titanium coated surfaces exhibited a pronounced broadband intensity increase in the spectral range between 1200 cm^− 1^ and 1420 cm^− 1^, centered near the titanium related vibrational mode at approximately 1370 cm^− 1^. This response was attributed to post-deposition oxidation of the sputtered titanium films, leading to the formation of mixed rutile and brookite titanium oxide phases that generate multiphoton vibrational modes detectable by Raman spectroscopy. These observations confirm chemical interaction at the coating resin interface while preserving the structural integrity of the polymer substrate forming the metamaterial-like architecture.

Diamond-like carbon coated substrates with metamaterial like architecture displayed well defined D and G bands at 1396 cm^− 1^ and 1589 cm^− 1^, respectively, confirming the presence of graphitic carbon domains with mixed *sp*^*2*^ and *sp*^*3*^ bonding configurations within an amorphous structure. Importantly, the primary vibrational peaks associated with the photopolymer substrates remained unshifted and intact across all coated specimens, indicating that the diamond-like carbon deposition process does not induce chemical degradation, crosslink scission, or molecular instability in the polymer matrix. Minor variations in spectral intensity observed between Accura^®^60 and Clear FLGPCL 02 substrates did not correspond to changes in vibrational frequencies, demonstrating consistent chemical behaviour across different resin systems supporting metamaterial like architecture.

A key outcome of this work is the demonstration that cost effective stereolithography platforms can serve as viable manufacturing route for producing coated microdevices with metamaterial-like architecture and complex geometries. The comparable coating conformity and molecular stability observed for both industrial and low cost stereolithography systems highlight the robustness and scalability of the proposed fabrication strategy for metamaterial like architectures requiring precise surface functionalization.

Overall, this study establishes a reliable and reproducible methodology for designing, fabricating and surface functionalisation of additively manufactured polymer substrates with metamaterial-like architecture through the integration of stereolithography and magnetron sputtering. The results demonstrate that high-quality and uniform titanium and diamond-like carbon coatings can be achieved without compromising geometric complexity or substrate chemical integrity. Future investigations will focus on quantitatively evaluating mechanical performance, tribological behaviour, wettability and biocompatibility of these coated metamaterial inspird architectures under realistic service conditions.

## Data Availability

Data will be made available on request.
